# Efficacy of psychotherapy in subthreshold depression patients: A protocol for an overview of systematic reviews and meta-analyses

**DOI:** 10.3389/fpubh.2022.1017907

**Published:** 2022-12-07

**Authors:** Xu Han, Jiaxin Li, Yajie Yang, Jiaxin Liu, Jinzi Zhang, Xiao Han, Abudurousuli Reyila, Zhizhong Liu, Pu Ge

**Affiliations:** ^1^School of Marxism, Liaoning University, Shenyang, China; ^2^Department of Psychology, Renmin University of China, Beijing, China; ^3^School of Nursing, Peking University, Beijing, China; ^4^Xiangya School of Nursing, Central South University, Changsha, China; ^5^School of Humanities and Social Sciences, Harbin Medical University, Harbin, China; ^6^Department of Pharmacy, The Fifth Affiliated Hospital of Sun Yat-sat University, Zhuhai, China; ^7^Xijing Hospital, Airforce Military Medical University, Xi'an, China; ^8^School of Finance and Trade, Liaoning University, Shenyang, China; ^9^Institute of Chinese Medical Sciences, University of Macau, Macao, Macao SAR, China

**Keywords:** subthreshold depression, psychotherapy, meta-analyses, systematic reviews, protocol

## Abstract

**Background:**

Subthreshold depression is a risk factor for major depression. Psychotherapy is a kind of intervention for subthreshold depression. There have been many systematic reviews synthesized the evidence for its effectiveness toward subthreshold depression. However, there is currently no overview of these systematic reviews.

**Objective:**

To undertake an overview of meta-analyses and systematic reviews to identify the efficacy of psychotherapy in subthreshold depression patients.

**Methods:**

We will search several databases such as PubMed, Embase, the Cochrane Library, Web of Science, PsycINFO, CNKI, WanFang and VIP database, for systematic reviews and meta-analyses on psychotherapy in subthreshold depression patients. The search timeline will be from inception up to August 2022. Two researchers will screen related studies back-to-back. We will include studies that evaluate the efficacy of psychotherapy in subthreshold depression patients. We will evaluate the methodological quality, the reporting quality and the quality of evidence for outcomes by AMSTAR-2, the PRISMA 2020 checklist and the GRADE grading system. We will present the results of the overview in alignment with the Preferred Reporting Items for Systematic Reviews and Meta-Analyses statement. The anticipated start and completion dates for this overview are 1 August 2022 and 30 December 2022, respectively.

**Results:**

From this study, we will evaluate the methodological quality and the level of evidence of the included systematic reviews and meta-analyses, and evaluate the efficacy of psychotherapy in patients with subthreshold depression.

**Implications:**

We will ascertain the efficacy of psychotherapy in subthreshold depression patients, to provide evidence to guide the treatment of subthreshold depression in the future.

**Registration number:**

Our research protocol has been registered with PROSPERO. The registration number of the protocol is CRD42021278871.

## Introduction

### Introduction of subthreshold depression

Subthreshold depression (SD) is a kind of less severe type of depression in which the individual does not meet the diagnostic threshold for major depressive disorder (MDD) in terms of frequency, severity, and/or duration of symptoms (1, 2). Currently, the most commonly used definition of SD in the scientific community, as described in Diagnostic and Statistical Manual 5^th^ Edition (DSM-5), is two or more symptoms of depressive disorder occurring simultaneously, one of which is depressed mood, for most or all of a period of at least 2 weeks. The symptoms must cause functional impairment and have a negative impact on the individual's life and may never meet the criteria for major depressive disorder or dysthymia (3, 4).

Studies based on population have found that SD is widely prevalence all over the world. For example, Stubbs et al. (5) described a 12-month SD prevalence of 2.5% and (6) reported a rate of 4.2%, based on 237,952 individuals and a sample of 3,514 residents of Izmir, Turkey, respectively. Relevant studies have also explored the epidemiological status of SD in people of different ages. For example, many studies have found that the prevalence of SD increases significantly after age 12 and rises permanently in mid-adolescence (14–16 years) (7–10). In adults, a systematic review of 19 studies estimated the prevalence of SD ranged from 3 to 10% in clinical and from 1 to 17% in community settings, respectively (1). In older people, the prevalence of SD is usually at least 2–3 times higher than that of MDD (11). In addition, studies have found that SD is associated with other diseases. For instance, According to the previous studies, the prevalence of SD among people with diabetes reached 12% (12), higher than the prevalence of depression (13).

Although SD has less severe symptoms than MDD (14), it causes a wide range of adverse outcomes (15). It has been found to be a risk factor for developing MDD and other psychiatric disorders, because of persistent depressive symptoms (16–18). For example, Lee et al. found that people with SD were about twice as likely to develop depression than non-depressed people (19). In addition, it is often an ineffective state with considerable psychological distress (14) and is also associated with increased mortality (17, 20) and significant economic costs (21). It is therefore important to identify effective methods for treating SD (22).

### Diagnosis and treatment of subthreshold depression

According to DSM-5, the diagnostic criteria for SD are “Depressed mood and at least one of the other eight symptoms of a Major Depressive episode, associated with clinically significant distress or deficit lasting at least 2 weeks, in individuals not meeting criteria for any other Depressive or Bipolar Disorder, and currently exhibiting no active or residual criteria for any Psychotic disorder, and does not meet the criteria of mixed anxiety or symptoms of depressive disorder.” In some studies, questionnaires were used to make a depression diagnosis. For example, Dirmaier et al. (23) categorized participants who scored between 5 and 8 on the Depression Screening Questionnaire (DSQ) as SD (23).

SD treatment aims to decrease or avoid the negative consequences associated with this depressive state and to act as a powerful tool for the prevention of MDD (24). There are multiple treatment strategies for SD, including pharmacotherapy, electroacupuncture, “Watchful waiting”, “Patient preferences”, and so on.

Pharmacotherapy for SD is mainly with antidepressants such as tricyclics, 5-hydroxytryptamine, reuptake inhibitors, etc. (25). The effectiveness of pharmacotherapy for SD is varied (26). Electroacupuncture has been extensively applied to the treatment of psychiatric disorders including depression (27), as it modulates the hypothalamic-pituitary-adrenal axis (28) and restores hippocampal CA1 synaptic plasticity by adjusting 5-hydroxytryptamine system receptor levels, thereby exerting antidepressant activity and improving depression-like behavior (29). “Watchful waiting” requires systematic monitoring, including assessment of symptoms, course, and impact, which may be appropriate for patients with good social support, no personal or family history of depressive disorder, and who refuse medication and/or psychotherapy even after explanation of risks and available treatment options (30). “Patient preferences” refers to educating patients about their disease and treatment options so that both physicians and patients can make shared decisions. A recent review has shown its benefits regarding adherence, treatment satisfaction, and outcomes (15). In addition to the therapies mentioned above, psychotherapy is becoming an increasingly important treatment option for SD. Currently psychotherapy is the intervention that has shown the strongest evidence of being the first-line treatment (31, 32). What's more, other studies suggest that, for comorbid SD, such as both depression and glycemic control, the preferred treatment would also be psychotherapy (33).

### Psychotherapy

Psychotherapy is the establishment of a relationship with the patient through structured and purposeful contact and the use of a range of specific techniques to improve the patient's psychological state (34), which has contributed greatly to the treatment of SD.

According to different studies, psychotherapy is usually categorized as follows: psychodynamic therapies, cognitive and behavioral therapies (CBT), humanistic therapies, systemic therapies (35) and interpersonal psychotherapy (IPT) (36). Each category can also be divided into different types, such as cognitive and behavioral therapies, which contain web-based, bibliotherapy-based and telephone-based cognitive behavioral therapy, and so on (25).

Psychodynamic therapies includes many approaches influenced by Freud's psychoanalysis, such as transfer-focused psychotherapy (TFP) and mentalization-based therapy (37). CBT is a form of talking therapy that aims to recognize and challenge non-adaptive beliefs and behaviors and try to develop distinct ways of thinking and behaving to improve the patient's mental and physical outcomes. Humanistic therapies adopt person-centered perspective, focusing on individual experiences and different needs of each patient (35). Systemic therapies, such as family therapy, assume that the patient's problems are situationally rather than personally derived (38). IPT is a highly structured, time-limited intervening that focuses on current salient relationship and interpersonal experiences (39). According to the available literature, mainstream psychotherapy for SD include CBT and IPT (21, 40, 41).

Several instrumental scales can be used to assess the effect of psychotherapy on SD. Generally speaking, Hamilton Rating Scale for Depression (HRSD) (42), the Kessler Screening Scale for Psychological Distress (K-6), Beck Depression Inventory scale (BDI), the Patient Health Questionnaire-9 (PHQ-9) and Center for Epidemiologic Studies Depression Scale (CES-D) can often be used as efficacy outcomes for SD (25). Moreover, Children's Depression Inventory (CDI), Children's Depression Rating Scale (CDRS) or Children's Depression Rating Scale-Revised (CDRS-R) can be used to measure the intervention effect in children (34, 43). Sometimes anxiety can be used as a secondary outcome indicator because anxiety problems have a high-comorbidity with depression (44).

Psychotherapy has significant effects on SD. Psychotherapy was proven to be more effective than care-as-usual for SD patients in a meta-analysis of 700 patients, reducing depressive symptoms and preventing episodes of severe depression (21). The long-term effect of psychological intervention on SD is to improve their psychological wellbeing, as well as to improve their physical and social functions (33). However, the efficacy may also be related to the specific treatments adopted and the characteristics of the patients. For instance, a systemic review including eight studies on the efficacy of Internet-based cognitive behavioral treatments (ICBT) discovered that ICBT intervention had better short-term efficacy for SD patients compared to controls, but it is inconclusive for long-term effects (44). And another meta-analytic review focusing on children and adolescents found a positive acute effect of psychological treatment for adolescents with SD, but this effect has not been found in children under 12 years of age (40). Recently, He et al. (45) conducted a network meta-analysis to compare and rank the efficacy of nonpharmacological interventions in adults with SD, and the study found that psychotherapy, especially CBT, may be the most effective nonpharmacological intervention to treat SD in adults. However, more RCTs examining the efficacy of different nonpharmacological interventions are needed in the future (14, 40, 41).

### Objective

Clinical and patient attention is increasingly focused on the treatment of SD by psychotherapy. There is a wide variety of psychotherapies available. With the popularization of systematic review methods, systematic reviews and meta-analyses of psychotherapies for SD are gradually increasing, but the quality are varied, so it is necessary to overview the systematic review. Meanwhile, there is no systemic overview to compare the therapeutic effects of different psychological interventions. Given the importance of psychotherapy in SD, and no overview has been conducted, this overview is expected to undertake a systematic overview to identify the efficacy of psychotherapy in SD patients and try to compare the efficacy of different kinds of psychotherapies.

## Materials and methods

### Study registration

We will follow the Preferred Reporting Items for Systematic Reviews and Meta-Analyses (PRISMA) statement for reporting our overview (46). Our research protocol has been registered with PROSPERO. The registration number of the protocol is CRD42021278871.

### Search strategy and selection criteria

We will search databases including PubMed, Embase, the Cochrane Library, Web of Science, PsycINFO, CNKI, WanFang and VIP database. The search terms are related to SD, systematic reviews and meta-analyses. To avoid omitting relevant studies, the interventions/controls of the studies will not be restricted during the literature search phase. We will screen systematic reviews and meta-analyses of interventions/controls that meet the inclusion criteria of this study during the literature screening phase. The search timeline will be from inception up to August 2022. Studies in Chinese or English will be included in this overview. As an example, the Search strategy of Pubmed database is shown in Table 1. Search strategy of other databases can be seen in Supplementary Table 1.

**Table 1:** The search strategy of Pubmed.

**Database**	**Search strategy**
PubMed	#1	(depression[mh]) OR (depression*[Title/Abstract]) OR (Depressive Symptom*[Title/Abstract])
	#2	(Depressive Disorder[mh]) OR (Depressive Disorder*[Title/Abstract]) OR (Depressive Disease[Title/Abstract]) OR (Depressive Neuroses[Title/Abstract]) OR (Depressive Neurosis[Title/Abstract]) OR (Depressive Syndrome*[Title/Abstract]) OR (Melancholia*[Title/Abstract])
	#3	(Depressive Disorder, Treatment-Resistant[mh]) OR ((Treatment Resistant[Title/Abstract]) AND (Depress*[Title/Abstract]))
	#4	(Depressive Disorder, Major[mh]) OR ((Depressi*[Title/Abstract]) AND (Major[Title/Abstract])) OR (Involutional Paraphrenia*[Title/Abstract]) OR (Involutional Psychoses[Title/Abstract]) OR (Involutional Psychosis[Title/Abstract]) OR ((Melancholia[Title/Abstract]) AND (Involutional[Title/Abstract]))
	#5	((bipolar[Title/Abstract]) AND (disorder*[Title/Abstract])) OR (bipolar illness[Title/Abstract]) OR (bipolar psychosis[Title/Abstract]) OR (manic depressive[Title/Abstract]) OR (manio depressive psychosis[Title/Abstract]) OR (depressive psychosis[Title/Abstract]) OR (dysphoria[Title/Abstract]) OR (unipolar disorder[Title/Abstract]) OR (melancholia[Title/Abstract]) OR (melancholic syndrome[Title/Abstract]) OR (melancholy[Title/Abstract]) OR (minor depressive episode[Title/Abstract]) OR (cothymia[Title/Abstract]) OR ((mourning[Title/Abstract]) AND (syndrome[Title/Abstract])) OR (Perry* syndrome[Title/Abstract]) OR (premenstrual dysphoric disorder[Title/Abstract]) OR (pseudodementia[Title/Abstract]) OR (pseudo dementia[Title/Abstract]) OR (seasonal affective disorder[Title/Abstract])
	#6	(brief recurrent[Title/Abstract]) OR (minor[Title/Abstract]) OR (subcase[Title/Abstract]) OR (subclinical[Title/Abstract]) OR (subthreshold[Title/Abstract]) OR (subsyndromal[Title/Abstract]) OR (subdiagnostic [Title/Abstract]) OR (sub-clinical[Title/Abstract]) OR (sub-threshold[Title/Abstract]) OR (sub-syndromal[Title/Abstract]) OR (sub-diagnostic [Title/Abstract])
	#7	(Meta-Analysis[Publication Type]) OR (Systematic Review[Publication Type]) OR (Systematic Review[Title/Abstract]) OR (meta-analysis[Title/Abstract]) OR (meta[Title/Abstract])
	#8	#1 OR #2 OR #3 OR #4 OR #5
	#9	#6 AND #8
	#10	#7 AND #9

#### Types of studies

Systematic reviews and meta-analyses of RCTs that examine the use of psychotherapy in SD patients will be included.

#### Types of participants

The participants had SD diagnosed according to any authoritative diagnostic criteria, no restrictions on age, sex, race, the source of cases, or onset time. Since SD is also found in minors, and there are also clinical studies and systematic reviews/meta-analyses on the treatment of SD in minors, in this overview, we will include relevant studies on the treatment of SD in minors.

#### Types of interventions

Systematic reviews and meta-analyses comparing one psychological intervention with another or either of the control conditions for patients with SD will be included. For psychotherapy, CBT, behavioral therapy (BT), cognitive therapy (CT), IPT, problem-solving therapy (PST), play therapy, supportive counseling, psychodynamic therapy (DYN) and family therapy will be included regardless of their treatment session and duration (34). In terms of control conditions, waiting-list control (WL), non-treatment control, treatment as usual (TAU) and (psychological or pill) placebo will be included.

#### Outcomes

The primary outcome of efficacy will be defined as mean overall change on continuous depression severity scales. Included systematic reviews or meta-analyses will be required to report at least one of the following indicators: Scores of HRSD, CDRS, CDRS-R, CES-D, BDI, the 9-item patient health questionnaire (PHQ-9), or the Kessler screening scale for psychological distress (K-6) (25).

The secondary indicators of this study are as follows. Quality of life/functioning improvement can be another indicator of psychotherapy. QoL/functioning an be captured by the mean overall change in functioning improvement scales or quality of life scales.

Suicide-related outcomes and anxiety can also be used as outcomes of psychological interventions. The former can be measured with the Suicidal Ideation Questionnaire-Junior High School version (47), and the latter can be measured with Hamilton Anxiety Scale Score (48).

#### Study screening process

**Figure 1:**
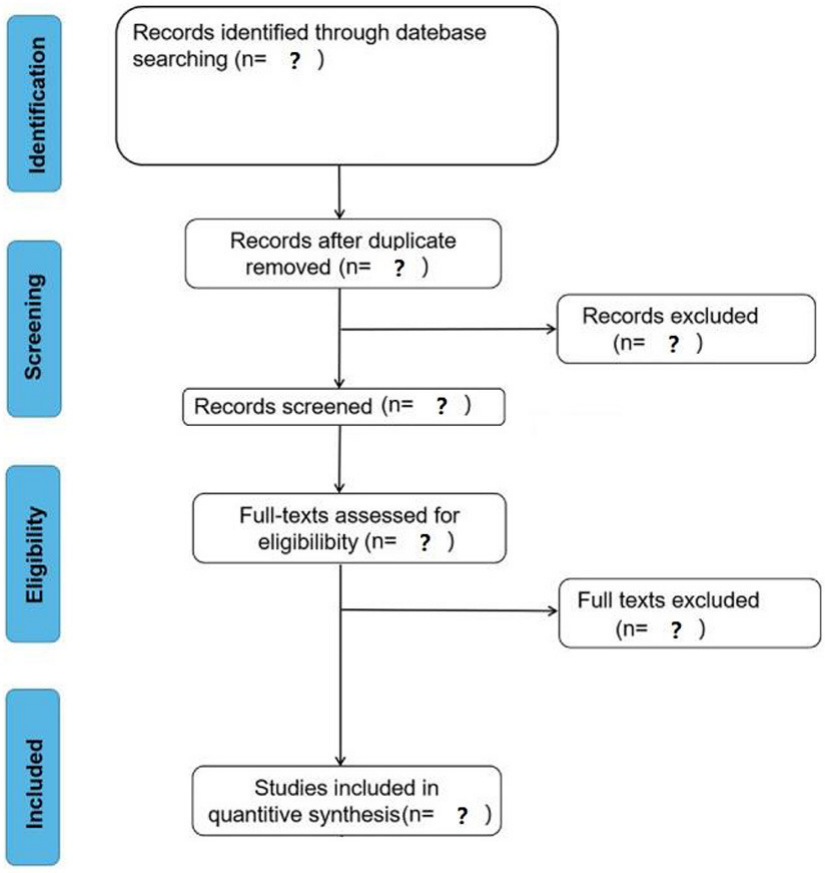
Schematic diagram of the screening process.

The screening process of the studies can be divided into four stages: deduplication, primary screening (screening studies based on titles and abstracts), secondary screening (screening studies based on full text), and cross-checking. After the studies are deduplication, two researchers will screen the eligible studies independently by reading the title, abstract and full text according to the inclusion and exclusion criteria. After the rescreening is completed, they will conduct a cross-check stage. If there is any disagreement, they will negotiate with the third researcher. The anticipated study screening process is shown in Figure 1.

### Data screening and extraction

The basic data will be independently extracted by two researchers according to the pre-designed data extraction table, including first author, publication year, intervention/control measures, the efficacy outcomes, etc. If the included literature was incomplete, we would contact the original researcher to obtain the required study data.

### Quality assessment

Two experienced investigators will conduct back-to-back quality evaluation of the included studies. If there is disagreement, another experienced investigator will be invited to join the discussion and finally reach an agreement.

A Measure Tool to Assess Systematic Reviews-2 (AMSTAR-2) (49) and the PRISMA 2020 checklist (46) will be used to evaluate the methodological quality of the included studies. As a recently developed methodological quality assessment tool for systematic reviews, AMSTAR-2 includes 16 items, which involve the whole process of systematic review topic selection, design, registration, data extraction, data statistical analysis and discussion. This tool classifies the methodological quality of systematic reviews into four levels: high, moderate, low, and very low. The PRISMA 2020 checklist includes a total of 27 items. When grading the included literature, 1 point is given for the item that is fully reported, 0.5 point for the partially reported item, and 0 point for the unreported item. The full score is 27 points. The literature with a score of < 15 points has relatively serious information defects, the literature with a score of ≥ 15 points and < 21 points has certain defects, and the report quality of the literature with a score of more than 21 points is relatively good.

The Grades of Recommendations Assessment, Development and Evaluation (GRADE) grading system of evidence will be used to assess the quality of evidence for outcomes (50, 51). The GRADE system, as a set of evidence rating system, includes five downgrading factors including study limitation, inconsistency, immediacy, imprecision and publication bias. It grades the quality of evidence into four levels: high, moderate, low, and very low.

### Data synthesis

A qualitative analysis will be performed first. The characteristics of the original studies included in each systematic reviews and the results of the meta-analysis will be summarized and concluded.

Secondly, a network meta-analysis will be performed by R (Version 4.0.3) with suitable packages such as gemtc.

The mean change and standard deviation of the outcome measures from baseline to the endpoint will be used to calculate the effect size (ES) of different kinds of psychotherapy. The ES will be assessed with standardized mean difference (SMD) because several measures of SD are reported in the included studies, but few of them are common to all trials. The *I*^2^ test will be used to assess heterogeneity when more than one studies are pooled, and the level of heterogeneity will be rated as low ( ≤ 50%), or high (>50%) (52). We will use a random-effects model or a fixed-effects model, which will depends on the heterogeneity of the studies included.

To combine direct and indirect evidence of SD, an NMA model will be conducted to compare the effects of different kinds of psychotherapy. If there are multiple groups or control groups classified as the same modality in a study according to our classification of intervention or control, we will split the article and conducted statistical analysis (53). The effects of different kinds of psychotherapy on SD will be compared using a network meta-analysis within the frequentist framework. We will calculate whether there will be a significant difference in the inconsistency model. If not, the consistency model will be selected. We will assess the assumption of consistency locally using the node-splitting method (54, 55). The surface under the cumulative ranking curve (SUCRA) will be used to separately rank each intervention (56). The larger the SUCRA value is, the better the rank. We will assess the efficacy of the different control groups as additional proof of transitivity by computing prepost treatment changes in the continuous depression severity score for the waitlist group, no treatment group, usual care group and active control group (57). A *p*-value < 0.05 was considered statistically significant.

When we perform sensitivity analyses, we will exclude studies having a high risk of bias and calculate the ranking of different kinds of psychotherapy when we included only low-risk studies. Publication bias will be examined funnel plots.

### Subgroup analyses

We will delivery the following subgroups analysis: (1) for the age of participants (e.g., Youth samples or Adult samples or Elderly samples); and (2) for the duration of psychotherapy (e.g., short-term treatment of six or fewer weeks or long-term treatment of more than 6 weeks); and (3) for the ample types of participants (e.g., Community-based samples or Primary care samples).

### Ethics and dissemination

This overview will not require the approval of an Ethics Committee because it will use published studies. We will publish results in a peer-reviewed journal.

## Discussion

Our overview will be a comprehensive synthesis of the existing systematic reviews and meta-analysis on the efficacy of psychotherapy in SD patients. To best of our knowledge, it will be the first overview in this filed.

In discussing our research, we plan to present the following sections: summary of key findings; comparison with other research and perspectives; implications for research and practice; interpretation of results; strengths and limitations; conclusions. We confirm that the results of this review will inform patients, physicians and clinical researchers about the reliability of the current evidence and the direction of future research.

## Author contributions

PG and ZL: conceptualisation, supervision, and writing—review and editing. XuH and JLi: investigation. PG: project administration. XuH, JLiu, YY, JLi, JZ, XiH, and AR: original draft. All authors read and approved the final manuscript.
