# Supplementary material for: Efficacy of psychotherapy in subthreshold depression patients: A protocol for an overview of systematic reviews and meta-analyses
Source: Front Public Health. 2022 Dec 7;10:1017907. doi: 10.3389/fpubh.2022.1017907 (PMC9768364; doi:10.3389/fpubh.2022.1017907)
Supplement: Supplementary file 1 [file Table_1.docx]

**Supplementary Table 1 Search Strategy of all databases**

| Database | Search strategy | |
| --- | --- | --- |
| Pubmed | #1  491,115 | (depression[mh]) OR (depression*[Title/Abstract]) OR (Depressive Symptom*[Title/Abstract]) |
|  | #2  137,606 | (Depressive Disorder[mh]) OR (Depressive Disorder*[Title/Abstract]) OR (Depressive Disease[Title/Abstract]) OR (Depressive Neuroses[Title/Abstract]) OR (Depressive Neurosis[Title/Abstract]) OR (Depressive Syndrome*[Title/Abstract]) OR (Melancholia*[Title/Abstract]) |
|  | #3  5,411 | ([Depressive Disorder, Treatment-Resistant](https://www.ncbi.nlm.nih.gov/mesh/68061218)[mh]) OR ((Treatment Resistant[Title/Abstract]) AND (Depress*[Title/Abstract])) |
|  | #4  86,909 | (Depressive Disorder, Major[mh]) OR ((Depressi*[Title/Abstract]) AND (Major[Title/Abstract])) OR (Involutional Paraphrenia*[Title/Abstract]) OR (Involutional Psychoses[Title/Abstract]) OR (Involutional Psychosis[Title/Abstract]) OR ((Melancholia[Title/Abstract]) AND (Involutional[Title/Abstract])) |
|  | #5  54,078 | ((bipolar[Title/Abstract]) AND (disorder*[Title/Abstract])) OR (bipolar illness[Title/Abstract]) OR (bipolar psychosis[Title/Abstract]) OR (manic depressive[Title/Abstract]) OR (manio depressive psychosis[Title/Abstract]) OR (depressive psychosis[Title/Abstract]) OR (dysphoria[Title/Abstract]) OR (unipolar disorder[Title/Abstract]) OR (melancholia[Title/Abstract]) OR (melancholic syndrome[Title/Abstract]) OR (melancholy[Title/Abstract]) OR (minor depressive episode[Title/Abstract]) OR (cothymia[Title/Abstract]) OR ((mourning[Title/Abstract]) AND (syndrome[Title/Abstract])) OR (Perry* syndrome[Title/Abstract]) OR (premenstrual dysphoric disorder[Title/Abstract]) OR (pseudodementia[Title/Abstract]) OR (pseudo dementia[Title/Abstract]) OR (seasonal affective disorder[Title/Abstract]) |
|  | #6  310,548 | (brief recurrent[Title/Abstract]) OR (minor[Title/Abstract]) OR (subcase[Title/Abstract]) OR (subclinical[Title/Abstract]) OR (subthreshold[Title/Abstract]) OR (subsyndromal[Title/Abstract]) OR (subdiagnostic [Title/Abstract]) OR (sub-clinical[Title/Abstract]) OR (sub-threshold[Title/Abstract]) OR (sub-syndromal[Title/Abstract]) OR (sub-diagnostic [Title/Abstract]) |
|  | #7  435,024 | (Meta-Analysis[Publication Type]) OR (Systematic Review[Publication Type]) OR (Systematic Review[Title/Abstract]) OR (meta-analysis[Title/Abstract]) OR (meta[Title/Abstract]) |
|  | #8  528,982 | #1 OR #2 OR #3 OR #4 OR #5 |
|  | #9  9,956 | #6 AND #8 |
|  | #10  415 | #7 AND #9 |
| Embase | #1  600,082 | 'depression'/exp |
|  | #2  623,139 | 'depression*':ab,ti OR 'depressive disease':ab,ti OR 'depressive disorder*':ab,ti OR 'depressive episode':ab,ti OR 'depressive illness':ab,ti OR 'depressive personality disorder':ab,ti OR 'depressive state':ab,ti OR 'depressive symptom':ab,ti OR 'depressive syndrome':ab,ti OR ('treatment resistant':ab,ti AND 'depress*':ab,ti) OR (' depressi*':ab,ti AND 'major':ab,ti) OR ('bipolar ':ab,ti AND 'disorder*':ab,ti) OR 'bipolar illness':ab,ti OR 'bipolar psychosis':ab,ti OR 'manic depressive':ab,ti OR 'manio depressive psychosis':ab,ti OR 'mano depressive syndrome':ab,ti OR 'depressive psychosis':ab,ti OR 'dysphoria':ab,ti OR 'melancholy':ab,ti OR 'melancholia':ab,ti OR 'unipolar disorder':ab,ti OR 'melancholic syndrome':ab,ti OR 'cothymia':ab,ti OR ('mourning':ab,ti AND 'syndrome ':ab,ti) OR 'Perry* syndrome':ab,ti OR 'premenstrual dysphoric disorder':ab,ti OR 'pseudodementia':ab,ti OR 'pseudo dementia':ab,ti OR 'seasonal affective disorder':ab,ti |
|  | #3  410,710 | 'brief recurrent':ab,ti OR 'minor':ab,ti OR 'subcase':ab,ti OR 'subclinical':ab,ti OR 'subthreshold':ab,ti OR 'subsyndromal':ab,ti OR 'subdiagnostic ':ab,ti OR 'sub-clinical':ab,ti OR 'sub-threshold':ab,ti OR 'sub-syndromal':ab,ti OR 'sub-diagnostic':ab,ti |
|  | #4  663,690 | 'meta analysis'/de OR 'meta analysis topic'/de OR 'systematic review'/de OR 'Systematic Review':ab,ti OR 'meta-analysis':ab,ti OR 'meta':ab,ti |
|  | #5  830,410 | #1 OR #2 |
|  | #6  14,971 | #3 AND #5 |
|  | #7  683 | #4 AND #6 |
| Cochrane Library | #1  14,311 | MeSH descriptor: [Depression] explode all trees |
|  | #2  105,065 | (Depression*):ti,ab,kw OR (Depressive Symptom*):ti,ab,kw OR (Depressive Disorder*):ti,ab,kw OR (Depressive Neurosis):ti,ab,kw OR (Depressive Neuroses):ti,ab,kw OR (Depressive Syndrome*):ti,ab,kw OR (Melancholia*):ti,ab,kw OR ((treatment resistant):ti,ab,kw AND (depress*):ti,ab,kw) OR ((depressi*):ti,ab,kw AND (major):ti,ab,kw) OR ((bipolar):ti,ab,kw AND ((disorder*):ti,ab,kw OR (psychosis):ti,ab,kw OR (psychoses):ti,ab,kw)) OR (Dysthymic Disorder*):ti,ab,kw OR (Dysthymia):ti,ab,kw OR ((Involutional):ti,ab,kw AND ((Psychosis):ti,ab,kw OR (Psychoses):ti,ab,kw OR (Melancholia):ti,ab,kw OR (Paraphrenia*):ti,ab,kw)) OR (((Psychoses,):ti,ab,kw OR (Psychoses):ti,ab,kw) AND ((Manic-Depressive):ti,ab,kw OR (Manic Depressive):ti,ab,kw)) OR (Adjustment Disorder*):ti,ab,kw OR ((Psychotic):ti,ab,kw AND (Disorder*):ti,ab,kw) |
|  | #3  23,519 | (brief recurrent):ti,ab,kw OR (minor):ti,ab,kw OR (subcase):ti,ab,kw OR (subclinical):ti,ab,kw OR (subthreshold):ti,ab,kw OR (subsyndromal):ti,ab,kw OR (subdiagnostic ):ti,ab,kw OR (sub-clinical):ti,ab,kw OR (sub-threshold):ti,ab,kw OR (sub-syndromal):ti,ab,kw OR (sub-diagnostic):ab,ti,kw |
|  | #4  105,065 | #1 OR #2 |
|  | #5  45 | #3 AND #4 in Cochrane Reviews |
| Web of Science | #1  949,925 | TS=(depression) |
|  | #2  808,943 | TI=(depression*) OR TI=(depressive disease) OR TI=(depressive disorder*) OR TI=(depressive episode) OR TI=(depressive illness) OR TI=(depressive personality disorder) OR TI=(depressive state) OR TI=(depressive symptom) OR TI=(depressive syndrome) OR (TI=(treatment resistant) AND TI=(depress*)) OR (TI=(depressi*) AND TI=(major)) OR (TI=(bipolar ) AND TI=(disorder*)) OR TI=(bipolar illness) OR TI=(bipolar psychosis) OR TI=(manic depressive) OR TI=(manio depressive psychosis) OR TI=(mano depressive syndrome) OR TI=(depressive psychosis) OR TI=(dysphoria) OR TI=(melancholy) OR TI=(melancholia) OR TI=(unipolar disorder) OR TI=(melancholic syndrome) OR TI=(cothymia) OR (TI=(mourning) AND TI=(syndrome)) OR TI=(Perry* syndrome) OR TI=(premenstrual dysphoric disorder) OR TI=(pseudodementia) OR TI=(pseudo dementia) OR TI=(seasonal affective disorder) OR AB=(depression*) OR AB=(depressive disease) OR AB=(depressive disorder*) OR AB=(depressive episode) OR AB=(depressive illness) OR AB=(depressive personality disorder) OR AB=(depressive state) OR AB=(depressive symptom) OR AB=(depressive syndrome) OR (AB=(treatment resistant) AND AB=(depress*)) OR (AB=(depressi*) AND AB=(major)) OR (AB=(bipolar ) AND AB=(disorder*)) OR AB=(bipolar illness) OR AB=(bipolar psychosis) OR AB=(manic depressive) OR AB=(manio depressive psychosis) OR AB=(mano depressive syndrome) OR AB=(depressive psychosis) OR AB=(dysphoria) OR AB=(melancholy) OR AB=(melancholia) OR AB=(unipolar disorder) OR AB=(melancholic syndrome) OR AB=(cothymia) OR (AB=(mourning) AND AB=(syndrome)) OR AB=(Perry* syndrome) OR AB=(premenstrual dysphoric disorder) OR AB=(pseudodementia) OR AB=(pseudo dementia) OR AB=(seasonal affective disorder) |
|  | #3  635,031 | TI=(brief recurrent) OR TI=(minor) OR TI=(subcase) OR TI=(subclinical) OR TI=(subthreshold) OR TI=(subsyndromal) OR TI=(subdiagnostic ) OR TI=(sub-clinical) OR TI=(sub-threshold) OR TI=(sub-syndromal) OR TI=(sub-diagnostic) OR AB=(brief recurrent) OR AB=(minor) OR AB=(subcase) OR AB=(subclinical) OR AB=(subthreshold) OR AB=(subsyndromal) OR AB=(subdiagnostic ) OR AB=(sub-clinical) OR AB=(sub-threshold) OR AB=(sub-syndromal) OR AB=(sub-diagnostic) |
|  | #4  745,429 | TI=(Systematic Review) OR TI=(meta-analysis) OR TI=(meta) OR AB=(Systematic Review) OR AB=(meta-analysis) OR AB=(meta) |
|  | #5  1,035,089 | #1 OR #2 |
|  | #6  15,224 | #3 AND #5 |
|  | #7  526 | #4 AND #6 |
| PsycINFO | S1 | SU depression |
|  | S2 | TI (depression*) OR TI (depressive disease) OR TI (depressive disorder*) OR TI (depressive episode) OR TI (depressive illness) OR TI (depressive personality disorder) OR TI (depressive state) OR TI (depressive symptom) OR TI (depressive syndrome) OR (TI (treatment resistant) AND TI (depress*)) OR (TI (depressi*) AND TI (major)) OR (TI (bipolar ) AND TI (disorder*)) OR TI (bipolar illness) OR TI (bipolar psychosis) OR TI (manic depressive) OR TI (manio depressive psychosis) OR TI (mano depressive syndrome) OR TI (depressive psychosis) OR TI (dysphoria) OR TI (melancholy) OR TI (melancholia) OR TI (unipolar disorder) OR TI (melancholic syndrome) OR TI (cothymia) OR (TI (mourning) AND TI (syndrome)) OR TI (Perry* syndrome) OR TI (premenstrual dysphoric disorder) OR TI (pseudodementia) OR TI (pseudo dementia) OR TI (seasonal affective disorder) OR AB (depression*) OR AB (depressive disease) OR AB (depressive disorder*) OR AB (depressive episode) OR AB (depressive illness) OR AB (depressive personality disorder) OR AB (depressive state) OR AB (depressive symptom) OR AB (depressive syndrome) OR (AB (treatment resistant) AND AB (depress*)) OR (AB (depressi*) AND AB (major)) OR (AB (bipolar ) AND AB (disorder*)) OR AB (bipolar illness) OR AB (bipolar psychosis) OR AB (manic depressive) OR AB (manio depressive psychosis) OR AB (mano depressive syndrome) OR AB (depressive psychosis) OR AB (dysphoria) OR AB (melancholy) OR AB (melancholia) OR AB (unipolar disorder) OR AB (melancholic syndrome) OR AB (cothymia) OR (AB (mourning) AND AB (syndrome)) OR AB (Perry* syndrome) OR AB (premenstrual dysphoric disorder) OR AB (pseudodementia) OR AB (pseudo dementia) OR AB (seasonal affective disorder) |
|  | S3 | TI (brief recurrent) OR TI (minor) OR TI (subcase) OR TI (subclinical) OR TI (subthreshold) OR TI (subsyndromal) OR TI (subdiagnostic ) OR TI (sub-clinical) OR TI (sub-threshold) OR TI (sub-syndromal) OR TI (sub-diagnostic) OR AB (brief recurrent) OR AB (minor) OR AB (subcase) OR AB (subclinical) OR AB (subthreshold) OR AB (subsyndromal) OR AB (subdiagnostic ) OR AB (sub-clinical) OR AB (sub-threshold) OR AB (sub-syndromal) OR AB (sub-diagnostic) |
|  | S4 | TI (Systematic Review) OR TI (meta-analysis) OR TI (meta) OR AB (Systematic Review) OR AB (meta-analysis) OR AB (meta) |
|  | S5 | S1 OR S2 |
|  | S6 | S3 AND S5 |
|  | S7  259 | S4 AND S6 |
| CNKI | 1 | (SU=('心理'+'情绪'+'抑郁'+'忧郁'+'沮丧') OR TKA=('心理'+'情绪'+'抑郁'+'忧郁'+'沮丧')) AND TKA='阈下' AND TKA=('系统评价'+'meta分析'+'荟萃分析') |
| WanFang | 1 | (主题:("抑郁"OR"心理"OR"情绪"OR"忧郁"OR"沮丧") OR 题名或关键词:("抑郁"OR"心理"OR"情绪"OR"忧郁"OR"沮丧") OR 摘要:("抑郁"OR"心理"OR"情绪"OR"忧郁"OR"沮丧")) AND (题名或关键词:"阈下") AND (题名或关键词:("系统评价"OR"meta分析"OR"荟萃分析") OR 摘要:("系统评价"OR"meta分析"OR"荟萃分析")) |
| VIP | 3 | (M=(抑郁 OR 心理 OR 情绪 OR 忧郁 OR 沮丧) OR R=(抑郁 OR 心理 OR 情绪 OR 忧郁 OR 沮丧)) AND (M=阈下 OR R=阈下) AND (M=(系统评价 OR meta分析 OR 荟萃分析) OR R=(系统评价 OR meta分析 OR 荟萃分析)) |
